# Unveiling the Noncanonical Autophagy-Independent Role of *ATG7* and *ATG9B* in Head and Neck Squamous Cell Carcinoma (HNSCC)

**DOI:** 10.1155/2022/9253938

**Published:** 2022-10-10

**Authors:** Yibo Guo, Yiting Sun, Mingtao Chen, Yisheng Feng, Xu Zhang, Tong Ji, Zheqi Liu, Yu Zhang

**Affiliations:** ^1^Department of Oral Maxillofacial-Head Neck Oncology, Shanghai Ninth People's Hospital, Shanghai Jiao Tong University School of Medicine, Shanghai, China; ^2^College of Stomatology, Shanghai Jiao Tong University, National Center for Stomatology, National Clinical Research Center for Oral Diseases, Shanghai Key Laboratory of Stomatology, Shanghai, China; ^3^Department of Oral and Cranio-Maxillofacial Surgery, Shanghai Ninth People's Hospital, Shanghai Jiao Tong University School of Medicine, Shanghai, China

## Abstract

The role of autophagy in cancer remains elusive, and nontargeted autophagy inhibitors have limited therapeutic effects in HNSCC. Here, we systematically analyzed the correlation of autophagy-related genes in HNSCC through TCGA and single-cell sequencing data (GSE103322). *ATG9B* and *ATG7* were found to have noncanonical autophagy-independent functions in HNSCC. Specifically, *ATG9B* was a protective factor in HNSCC patients through downregulating cancer cell EMT, and *ATG7* was correlated with the immunosuppressive environment in HNSCC. Mechanistically, single-cell analysis revealed that *ATG9B* increased the epithelial phenotype of cancer cells but did not influence EMT signaling pathways. *ATG7* was strongly correlated with elevated immunosuppressive checkpoints like PD-1, PD-L1, and CTLA4 in HNSCC. Further single-cell analysis and multiple immunofluorescence colocalization analyses indicated that *ATG7* contributed to the high expression of PD-L1 in myeloid cells but not cancer cells. Collectively, our results revealed noncanonical autophagy-independent functions of autophagy-related genes. These results increase understanding of the intricacies of autophagy and may contribute to precision treatment using autophagy-targeted therapies.

## 1. Introduction

Macroautophagy (referred to throughout this article as autophagy) is a highly conserved catabolic process, which involves the formation of double-membraned vesicles known as autophagosomes that engulf cellular proteins and organelles for delivery to the lysosome. Autophagy is also highly involved in tumor initiation and progression; however, it remains controversial whether it plays a tumor-suppressive or a tumor-promoting role in different tumor types. It is now generally acknowledged that autophagy plays a larger role in tumor inhibition during tumorigenesis and malignant transformation. For example, mice with monoallelic deletion of the autophagy-related gene *beclin1* eventually develop spontaneous tumors [1]. Additionally, mice lacking Atg4 are more prone to chemically-induced fibrosarcoma [2]. However, autophagy may exert an opposite function in established tumors. It can protect tumor cells from metabolic stressors such as glucose and amino acid deficiency [3] as well as help cancer cells survive chemotherapy drugs and targeted therapy drugs [4, 5]. Aside from the canonical functions of autophagy in tumor progression, cumulative evidence shows that autophagy is also involved in other hallmarks of cancer like cancer metabolism [6], metastasis, and immune escape [7, 8] depending on the tumor type. The multifaceted effects of autophagy on tumors make targeting autophagy for cancer therapy a significant problem. Mining of the noncanonical functions of autophagy during cancer progression may increase understanding of the intricacies of autophagy and contribute to precision treatment using autophagy-targeted therapies.

Head and neck squamous cell cancer (HNSCC) is a common and aggressive malignancy with a poor 5-year survival rate of 45% [9], and it is closely correlated with overuse of tobacco and alcohol. The role of autophagy in HNSCC remains ambiguous, as it has been implicated in processes from inhibition to overactivation in HNSCC [10]. Despite success in inhibiting cancer cell growth through autophagy inhibition in vitro [11], in vivo curative effects of the nontargeted autophagy inhibitors hydroxychloroquine and 3-MA have been less successful. Furthermore, different autophagy-related genes play different roles beyond their functions in autophagy and autophagy-related membrane-trafficking pathways [12]. Understanding the specific roles of individual autophagy genes will contribute to more accurate autophagy-targeted therapies. In this study, we have explored the noncanonical functions of autophagy-related genes by analyzing The Cancer Genome Atlas (TCGA) database and single-cell HNSCC database (GSE103322). This study provides a new perspective on specific autophagy-related genes in HNSCC, and may aid in the development of more accurate autophagy-targeted therapies.

## 2. Methods

### 2.1. RNA-Sequencing Data from TCGA

The gene expression data, including the count and fragments per kilobase of transcript per million mapped reads (FPKM), and related clinical data of The Cancer Genome Atlas (TCGA) HNSC project were downloaded from the UCSC Xena online database (http://xena.ucsc.edu/). For further analysis, the FPKM data were transformed into transcripts per million reads (TPM). This study met the publication guidelines described by TCGA.

### 2.2. Differential Gene Expression Analysis

Identification of differentially expressed genes (DEGs) between different groups were performed using limma package. Log2FoldChange > 2 and adjust *P* value <0.01 were set as the threshold values for DEGs [13].

### 2.3. Functional Analysis of DEGs

The Gene Ontology (GO) and Kyoto Encyclopedia of Genes and Genomes (KEGG) analyses of correlated neighboring genes were conducted using the enrichGO and enrichKEGG functions of the clusterProfiler package, respectively [14]. GSEA analysis was performed using the GSEA function of clusterProfiler package. Adjusted *P* values (false-discovery rate [FDR]) lower than 0.05 were considered to indicate statistical significance. Gene set permutations were performed 1000 times for each analysis.

### 2.4. Tumor Purity Analysis

ESTIMATE is a method that uses gene expression signatures to infer the fraction of stromal and immune cells in tumor samples [15]. The tumor purity of TCGA samples was calculated using the ESTIMATE package in R.

### 2.5. Analysis of Immune Infiltration

TIMER (http://timer.cistrome.org/) is a reliable and intuitive tool for inferring immune infiltration levels from TCGA datasets [16]. The immune infiltration level scores of TCGA samples, including scores calculated by TIMER, CIBERSORT [17], MCPcounter [18], and EPIC [19], were downloaded from the TIMER database for further analysis.

### 2.6. Single-Cell Sequencing Data Analysis

Single-cell sequencing data from GSE103322 [20] was downloaded from Gene Expression Omnibus (GEO) database and had already been normalized by the researcher who deposited it. Cancer and noncancer cells were already labelled by the researcher. The matrix was combined in R and converted to a Seurat object using the Seurat R package [21]. To reduce dimensionality, principal component analysis was employed to summarize the resulting variably expressed genes, and then t-SNE dimensionality reduction (RunTSNE function) was used to further summarize principal components. In cancer cells, all the cells were divided into 13 clusters using FindNeighbors and FindClusters functions in Seurat. In noncancer cells, the clusters of each cell type were annotated based on expression of the following gene sets: CD4+ T cells (*CD4* and *IL7R*), Tregs (*CD4*, *FOXP3*, and *IL2RA*), CD8+ T cells (*CD8A* and *CD8B*), exhausted CD8+ T cells (*CD8A*, *CD8B*, *PDCD1*, *CTLA4*, and *LAG3*), myeloid cells (*TPSB2*, *CD1A*, *CD14*, *CD163*, *C1QA*, and *TREM1*), fibroblasts (*COL1A2* and *DCN*), NK cells (*NCAM1*, *KLRD1*, *KLRC1*, and *XCL1*), endothelial cells, (*PECAM1* and *VWF*), and B cells (*CD79A* and *CD19*).

### 2.7. Cell Culture

The human OSCC cell lines CAL27 cells were purchased from the American Type Culture Collection (ATCC, USA). All of these cells were maintained in Dulbecco's minimum essential medium (Invitrogen, Carlsbad, CA, USA) supplemented with 10% fetal bovine serum (FBS), 100 units/ml penicillin and 100 *μ*g/ml streptomycin incubated in a humidified atmosphere with 5% CO2 at 37°C.

### 2.8. Western Blot

Western blotting was performed as described previously [9]. The antibodies against the following proteins were used: *ATG7* (1 : 1000, 8558, Cell Signaling Technology), PD-L1 (1 : 1000, 13684, Cell Signaling Technology), *β*-actin (1 : 5000, 4970, Cell Signaling Technology), and *ATG9B* (1 : 1000, A7406, ABclonal). The immunoreactive bands were visualized using an Odyssey® Infrared Imaging System (Bioscience USA).

### 2.9. Immunofluorescence

The HNSCC patient paraffin sections were incubated with anti-ATG7, anti-PD-L1 and anti-CD-68 antibodies overnight at 4°C and then washed and incubated for 30 min with an Alexa Fluor 488 donkey anti-rabbit IgG (H+L) (Invitrogen, USA; 1 : 500) or an Alexa Fluor 549 donkey anti-mouse IgG (H+L) (Invitrogen, USA; 1 : 500) at room temperature in the dark. The nuclei were stained with 4′, 6-diamidino-2-phenylindole (DAPI; Invitrogen, USA; 1 : 1000) for 5 min. The cells were imaged using an Axio Vert. A1 microscope (Carl Zeiss, Germany). Prior to the use of the clinical materials for research purpose, patients' written informed consents and approval were obtained. The use of human specimens in this study was approved by the Institutional Research Ethics Committee of Shanghai Ninth People's Hospital.

### 2.10. Statistical Analysis and Data Visualization

Statistical analysis was performed using R (4.0.2). The Spearman correlation tests were performed to analyze the correlation among the expression of different autophagy related genes. Gene expression of *ATG7*/*ATG9B* was compared using the Wilcoxon rank-sum test. The association between the expression of *ATG7*/*ATG9B* and survival was analyzed using Cox regression models. The relationships between clinical parameters and the expression of *ATG7*/*ATG9B* were analyzed using the Wilcoxon rank-sum test. Correlation analyses between the expression of *ATG7* and tumor purity, the RNA expression of *CD274*, *PDCD1*, and *CTLA4*, or immune infiltration levels were also performed using the Spearman correlation tests. Results were visualized with the ggplot2, pheatmap, clusterProfiler, and Seurat packages.

## 3. Results

### 3.1. Different Clusters of Autophagy-Related Genes

**Figure 1:**
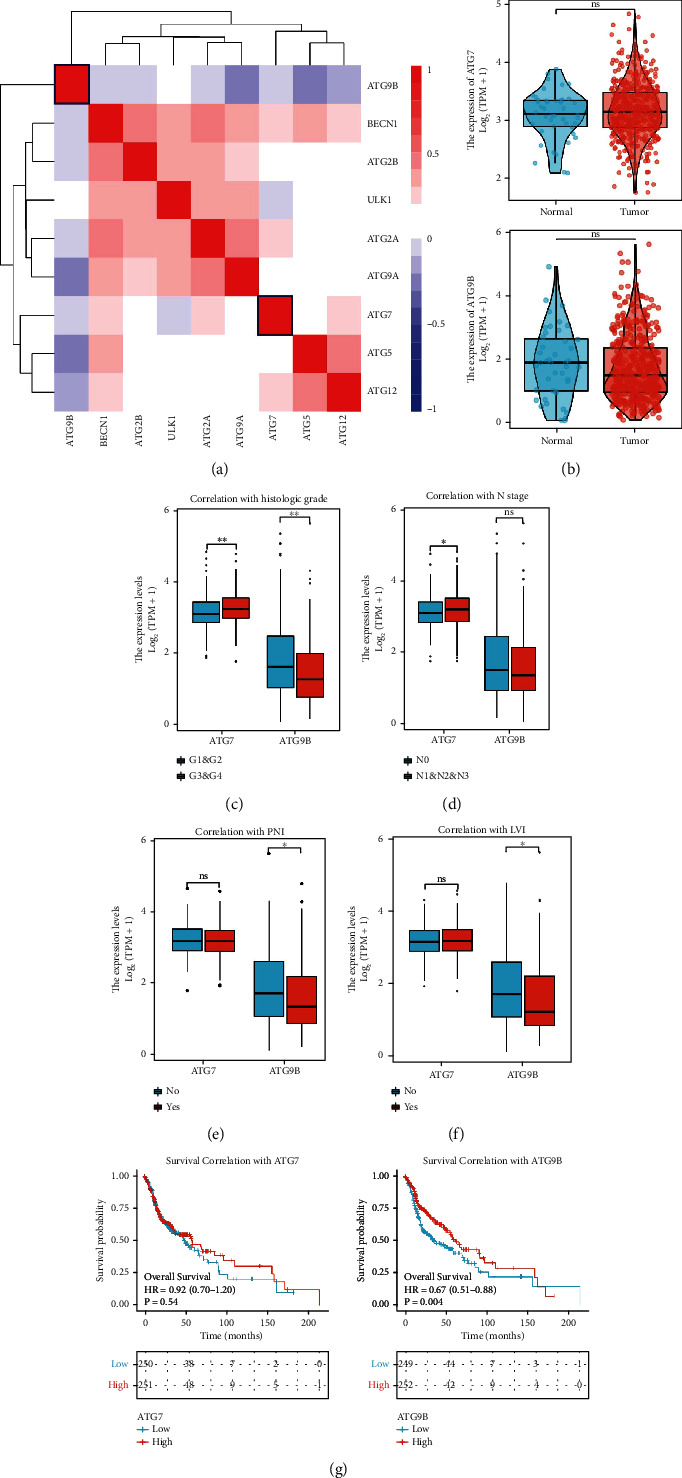
*ATG7* and *ATG9B* may play autophagy-independent roles in HNSCC. (a) Correlation of different autophagy-related genes with transcription level based on the TCGA database. (b) The mRNA levels of *ATG7* and *ATG9B* in HNSCC tumor and normal tissues. (c–f) The correlation between clinicopathological features and expression of *ATG7* and *ATG9B* in HNSCC patients. (g) Kaplan-Meier analysis of the mRNA expression of *ATG7* and *ATG9B* on the overall survival rate of HNSCC patients.

To assess the correlation between autophagy-related genes, we downloaded expression profiling datasets for 517 HNSCC patients in TCGA. We analyzed the correlations of the autophagy-related genes *BECN1*, *ULK1*, *ATG2A*, *ATG2B*, *ATG5*, *ATG7*, *ATG9A*, *ATG9B*, and *ATG12*. The results showed that *BECN1*, *ULK1*, *ATG2A*, *ATG2B*, and *ATG9A* had stronger correlation in transcriptomes (thus we named it cluster A), while *ATG5* and *ATG12* were strongly correlated with each other (thus we named it cluster B) (Figure 1(a)). Strikingly, *ATG7* had a weaker correlation with both cluster A and B, and *ATG9B* was the only autophagy-related gene negatively correlated with the other clusters, suggesting it might have other autophagy-independent functions.

These correlations were in accordance with the protein functions in autophagy. *BECN1*, *ULK1*, *ATG2A*, *ATG2B*, and *ATG9A* were more involved in autophagy initiation and phagophore formation, while *ATG5* and *ATG12* were involved in the elongation of phagophores. Therefore, we further investigated the potential autophagy-independent roles of *ATG7* and *ATG9B*. Compared to normal tissues, the *ATG7* mRNA level was slightly upregulated, and the *ATG9B* mRNA level was slightly downregulated in HNSCC tissues in the TCGA database (Figure 1(b)). However, both results were not significantly different. We then analyzed the correlation between the mRNA expression of *ATG7* and *ATG9B* and the clinicopathological features of HNSCC patients in the TCGA database. Higher *ATG7* expression was correlated with high histologic grade (*P* < 0.01) and lymph node metastasis (*P* < 0.05) (Figures 1(c)–1(f)). Strikingly, higher *ATG9B* expression was correlated with lower histologic grade (*P* < 0.01), lower chance of perineural invasion (*P* < 0.05), and lower chance of lymphatic vascular invasion (*P* < 0.05) (Figures 1(c)–1(f)). Kaplan-Meier analysis (KM) showed that HNSCC patients with higher *ATG9B* had better overall survival rates (OS) (HR = 0.67, *P* = 0.004) (Figure 1(g)). Collectively, these results indicated that *ATG7* and *ATG9B* might play autophagy-independent roles in HNSCC. Specifically, *ATG7* might play a role in tumor promotion, while *ATG9B* might be a tumor suppressor.

### 3.2. *ATG9B* Was Negatively Correlated with HNSCC Epithelial Mesenchymal Transition (EMT) in TCGA

**Figure 2:**
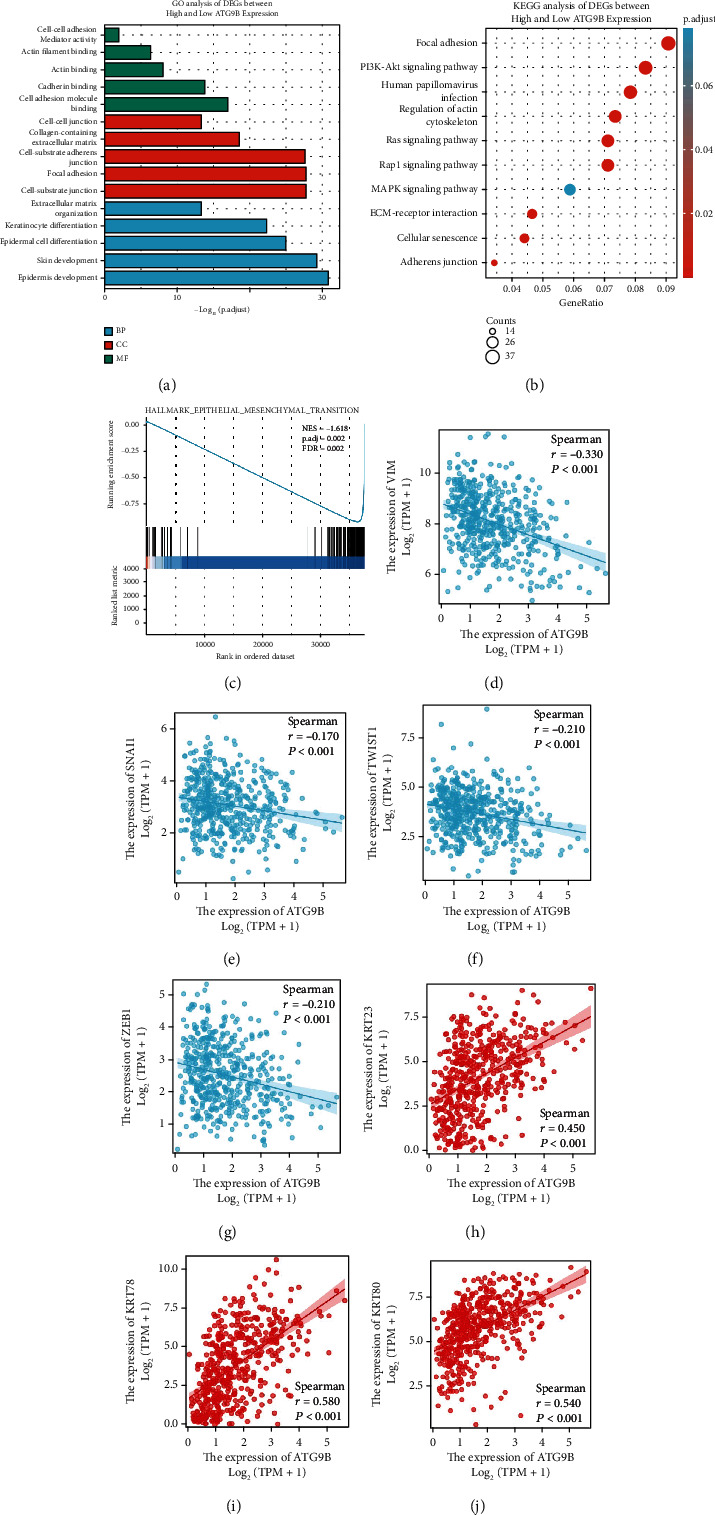
*ATG9B* was negatively correlated with HNSCC EMT in TCGA. (a) GO analysis of different expression genes with a fold change of >1.5 or <0.667 and an FDR < 0.05 based on *ATG9B* mRNA expression. (b) KEGG analysis of different expression genes with a fold change of >1.5 or <0.667 and an FDR < 0.05 based on *ATG9B* mRNA expression. (c) GSEA analysis showed that *ATG9B* was negatively correlated with HNSCC EMT. (d–g) The expression of *ATG9B* was negatively correlated with EMT marker genes. (h–j) The expression of *ATG9B* was strongly correlated with the keratin family.

The transcriptomes of HNSCC patients from the TCGA database were divided into 2 groups according to *ATG9B* mRNA expression. Differential gene expression with a fold change >1.5 or <0.667 and an FDR < 0.05 were identified and underwent GO analysis. GO analysis showed that *ATG9B* was highly involved in the EMT process including cell-cell adhesion mediator activity, cadherin binding, and extracellular matrix organization (Figure 2(a)). KEGG analysis showed similar results like focal adhesion, regulation of the actin cytoskeleton, ECM-receptor interaction, and adherens junction (Figure 2(b)). Gene Set Enrichment Analysis (GSEA) indicated that *ATG9B* was negatively correlated with HNSCC EMT (NES = 1.618, FDR = 0.002) (Figure 2(c)). We further found that *ATG9B* was negatively correlated with the EMT marker genes SNAIL (*r* = −0.017, *P* < 0.001), TWIST (*r* = −0.210, *P* < 0.001), ZEB1 (*r* = −0.210, *P* < 0.001), and VIM (*r* = −0.330, *P* < 0.001) (Figures 2(d)–2(g)). Furthermore, *ATG9B* was strongly correlated with the keratin family, especially KRT23 (*r* = 0.450, *P* < 0.001), *KRT78* (*r* = 0.580, *P* < 0.001), and *KRT80* (*r* = 0.540, *P* < 0.001) (Figures 2(h)–2(j)). These three KRT genes had much lower expression in normal tissues compared to HNSCC (Figure S1). These results showed that cancer cells with higher *ATG9B* expression had a phenotype closer to epithelial cells, which might partially explain the negative correlation between *ATG9B* expression and PNI and lymphatic vascular invasion in HNSCC. Moreover, we explored the protein levels of *ATG9B* in human HNSCC tissues. As expected, there was significantly lower *ATG9B* protein levels in tumor tissues compared to adjacent normal tissues (Supplementary Figure 2).

Together, these results indicate that high *ATG9B* expression is positively correlated with the epithelial cell phenotype of HNSCC cells, which might contribute to an impaired EMT phenotype.

### 3.3. Verification of the Function of *ATG9B* in HNSCC through the Single-Cell Database

**Figure 3:**
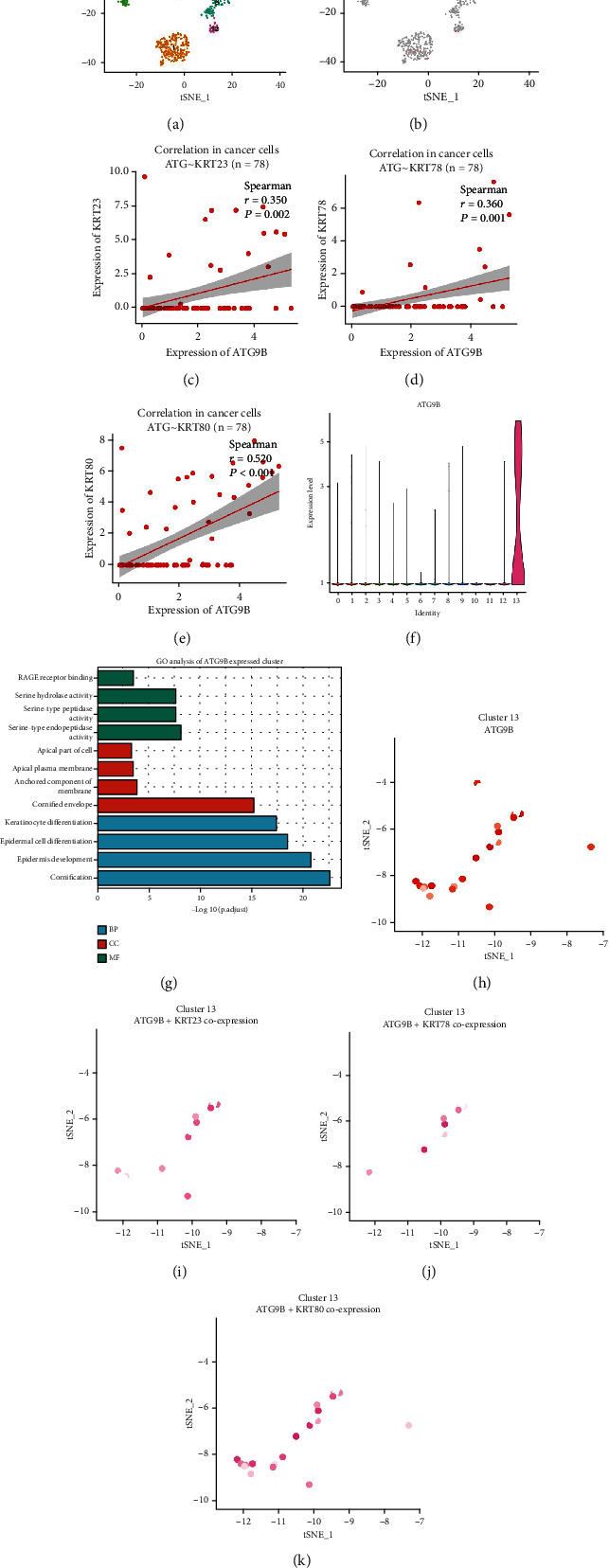
*ATG9B* was negatively correlated with HNSCC EMT in the single-cell database (GSE103322). (a) Different clusters of cancer cells in GSE103322. (b) Expression of ATG9B in cancer cells. (c–e) Correlation of *ATG9B* with *KRT23*, 78, and 80 in cancer cells. (f) *ATG9B* was mainly expressed in cluster 13 of cancer cells. (g) GO analysis of highly expressed genes in cluster 13 cancer cells. (h–k) *ATG9B* was coexpressed with *KRT23*, 78, and 80 in cluster 13 cancer cells.

To further determine the distribution of *ATG9B* in HNSCC and verify its function in specific cell types, we mined the GSE103322 single-cell database (Figure 3(a)). We found that *ATG9B* was mainly expressed in cancer cells and not in immune and stromal cells (Figure 3(b)). Then, we verified the correlation between *ATG9B* and the KRT family. As expected, *ATG9B* was positively correlated with KRT23 (*r* = 0.350, *P* = 0.002), *KRT78* (*r* = 0.360, *P* = 0.001), and *KRT80* (*r* = 0.520, *P* < 0.001), which again proved that cancer cells with higher expression of *ATG9B* have a phenotype similar to the epithelial cell (Figures 3(c)–3(e)). Then, we focused on the cell cluster with high expression of *ATG9B*. The violin figure shows that *ATG9B* was mainly expressed in cancer cell cluster 13 (Figure 3(f)). We further identified the highly expressed genes in cluster 13 and ran a GO analysis. The results indicated that cancers in cluster 13 had more epidermal development, epidermal cell differentiation, and keratinocyte differentiation (Figure 3(g)). Finally, we identified the cancer cells in cluster 13 and the single cells expressing both *ATG9B* and *KRT23*, *KRT78*, or *KRT80* (Figures 3(i)–3(k)). Collectively, these results indicate that high *ATG9B* expression is positively correlated with the epithelial phenotype of single cancer cells through mining the HNSCC single-cell database.

### 3.4. *ATG7* Was Involved in the Tumor Immune Microenvironment (TIME) in HNSCC, but Did Not Function in Cancer Cells

**Figure 4:**
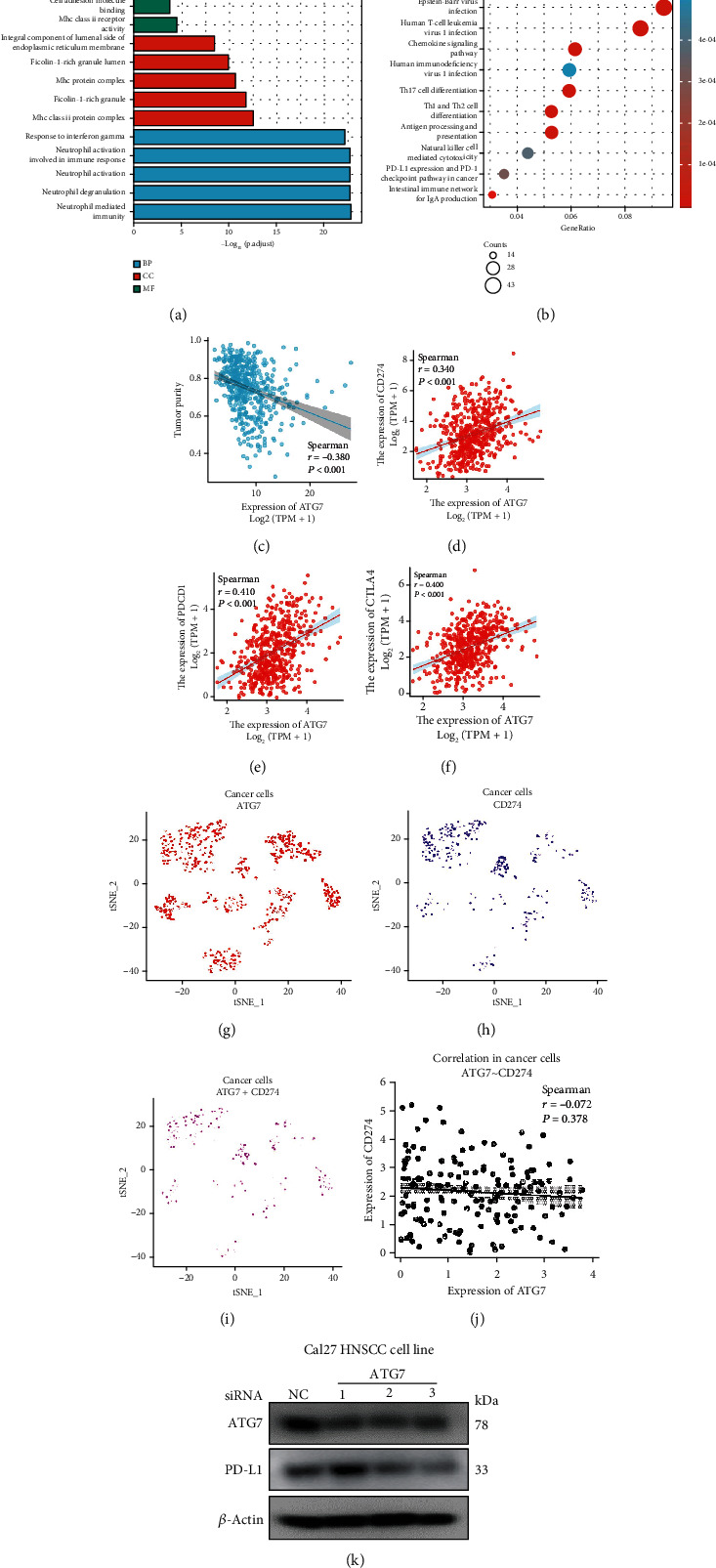
*ATG7* was involved in the tumor immune microenvironment (TIME) in HNSCC, but did not function in cancer cells. (a) GO analysis of different expression genes with a fold change >1.5 or <0.667 and an FDR < 0.05 based on the mRNA expression of *ATG7* in HNSCC in the TCGA database. (b) KEGG analysis of different expression genes with a fold change >1.5 or <0.667 and an FDR < 0.05 based on the mRNA expression of *ATG7* in HNSCC in the TCGA database. (c) The correlation between *ATG7* expression and tumor purity in HNSCC in the TCGA database. (d–f) *ATG7* was positively correlated with PD-L1, PD1, and CTLA4. (g–j) *ATG7* was not correlated with PD-L1 in cancer cells in GSE103322. (k) Knockdown of *ATG7* did not affect the expression of PD-L1 in the Cal27 HNSCC cell line.

The transcriptomes of HNSCC patients in TCGA were divided into 2 groups according to *ATG7* mRNA expression. Differential gene expression with a fold change of >1.5 or <0.667 and an FDR < 0.05 was identified and underwent GO analysis. The GO items showed that *ATG7* was highly involved in the tumor immune microenvironment in HNSCC, including chemokine activity, MHC protein procession, and neutrophil activation (Figure 4(a)). KEGG analysis was conducted, and the top 10 items were correlated with immune response (Figure 4(b)). However, the “PD-L1 expression and PD-1 checkpoint pathway in cancer” item caught our attention. We then analyzed the relationship between expression of *ATG7* and tumor purity in HNSCC using the TCGA database. *ATG7* was negatively correlated with tumor purity (*r* = −0.038, *P* < 0.001), suggesting a positive correlation between high ATG expression and high infiltrating immune cells in HNSCC tumors (Figure 4(c)). The increased immune cells in TIME suggested that *ATG7* might enhance the antitumor immune response. However, further analysis of the correction between *ATG7* and immunosuppressive checkpoints showed that high expression of *ATG7* was correlated with high expression of PD-L1 (*r* = 0.340, *P* < 0.001), PD-1 (*r* = 0.410, *P* < 0.001), and CTLA4 (*r* = 0.400, *P* < 0.001) in HNSCC, which implied that ATG might be involved in immunosuppression in HNSCC (Figures 4(d)–4(e)).

To further determine whether *ATG7* exerts its immunosuppressive functions in cancer cells or immune cells, we analyzed the single-cell data from GSE103322. It is generally thought that cancer cells express high levels of PD-L1, thus suppressing the anticancer function of the immune system. We identified the cancer cells expressing both *ATG7* and PD-L1 but found no correlation between *ATG7* and PD-L1 in cancer cells (Figures 4(g)–4(j)). Finally, we found that the protein level of PD-L1 was not influenced by knockdown of *ATG7* in the Cal27 HNSCC cell line. Collectively, these data showed that *ATG7* was involved in TIME in HNSCC, and it potentially did not function in cancer cells.

### 3.5. High *ATG7* Expression Was Correlated with High PD-L1 Expression in Noncancer Cells in HNSCC

**Figure 5:**
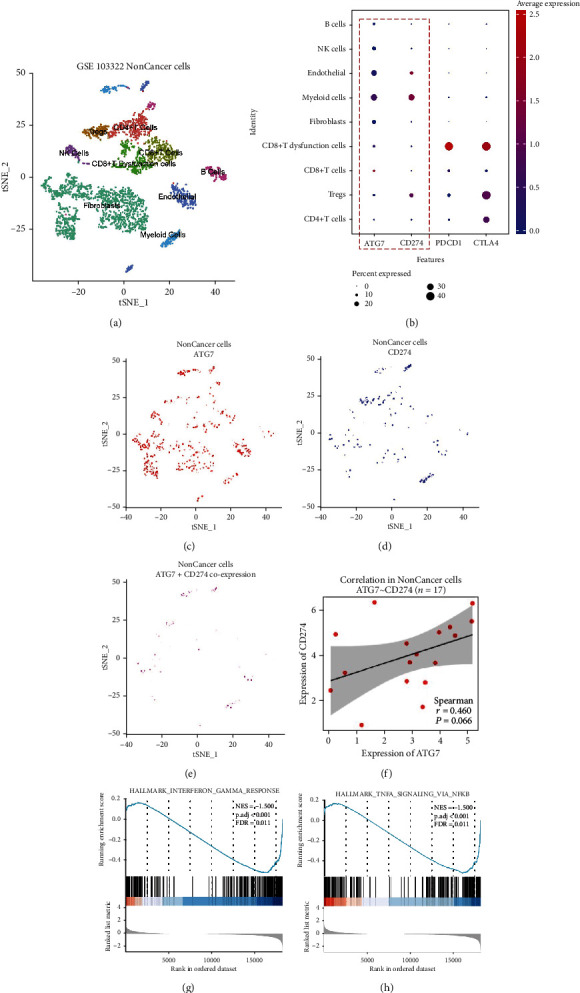
High *ATG7* expression was correlated with high PD-L1 expression in noncancer cells in HNSCC in GSE103322. (a) Different clusters of noncancer cells in GSE103322. (b) *ATG7* had a similar distribution to PD-L1 but not PD1 or CTLA4 in noncancer cells. (c–f) Cells expressing both *ATG7* and PD-L1 were identified, and *ATG7* had a positive correlation with PD-L1. (g–h) Genes highly expressed in cells expressing both *ATG7* and PD-L1 underwent GSEA analysis.

To further determine the function of *ATG7* in HNSCC TIME, we further explored the correlation of *ATG7* and noncancer cells using GSE103322 (Figure 5(a)).  Figure 5(b) shows the expression of ATG7, PD-L1, PD1, and CTLA4 in noncancer cells. We found that *ATG7* had a similar distribution to PD-L1 but not PD1 or CTLA4 in noncancer cells. Cells expressing both *ATG7* and PD-L1 were identified, and *ATG7* had a positive correlation with PD-L1 (*r* = 0.460, *P* = 0.0660) (Figures 5(c)–5(f)). Furthermore, genes highly expressed in cells expressing both *ATG7* and PD-L1 underwent GSEA analysis. Results showed that these cells had a downregulated response to interferon*γ* (NES = −1.500, FDR = 0.011) and TNF*α* (*r* = 0.46, *P* = 0.066) (Figures 5(g)–5(h)). These results further proved that *ATG7* exerted its immunosuppressive function in noncancer cells through regulating PD-L1 expression.

### 3.6. *ATG7* Was Correlated with Myeloid Cells in HNSCC TIME

**Figure 6:**
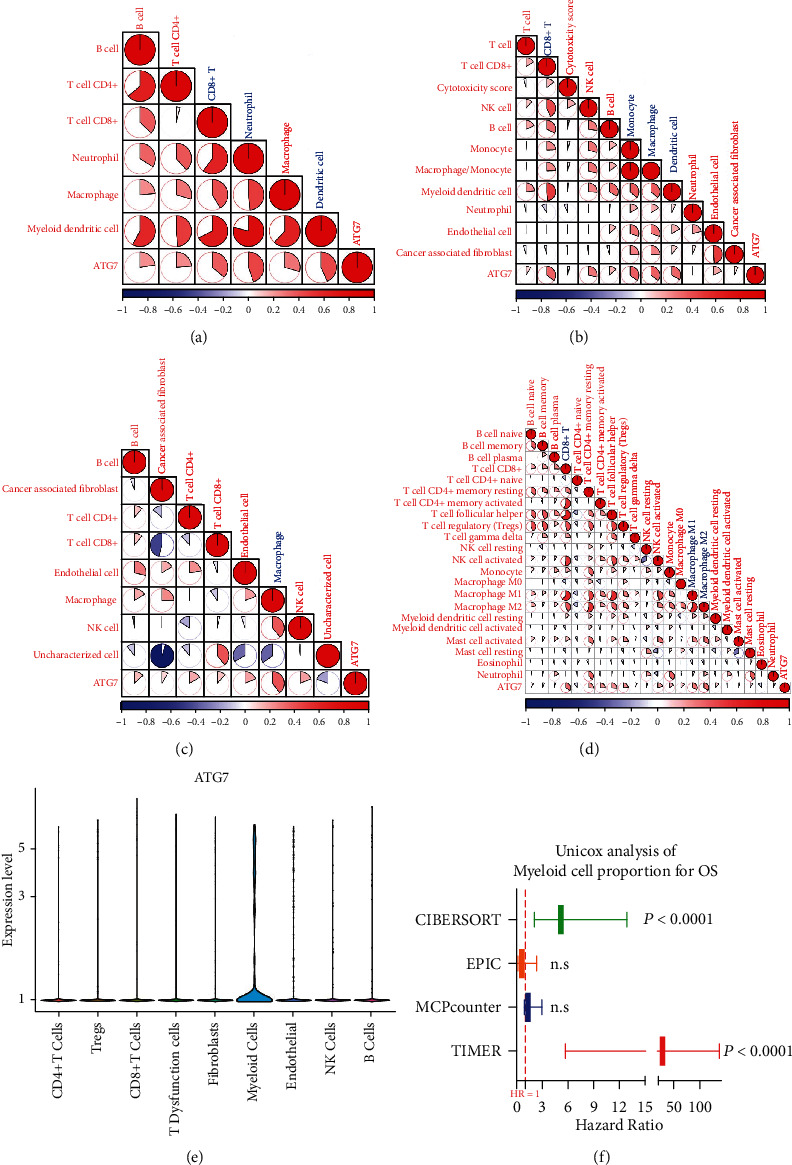
*ATG7* was correlated with high PD-L1 expression in myeloid cells in the HNSCC TIME. (a–d) Correlation between *ATG7* and noncancer immune cells in the TIMER, MCPcounter, EPIC, and CIBERSORT databases according to TCGA. (e) *ATG7* was mainly expressed in myeloid cells in GSE103322. (f) The high proportion of myeloid cell in tumors was correlated with poor overall survival-rates in the CIBERSORT and TIMER databases.

**Figure 7:**
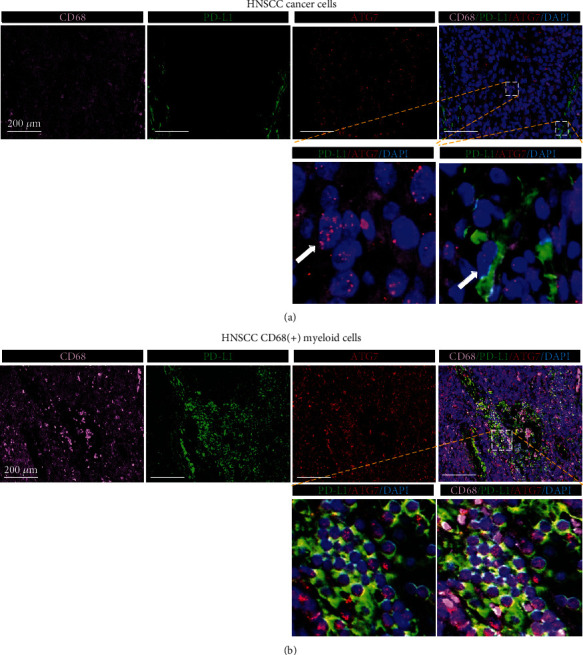
*ATG7* was coexpressed with PD-L1 in CD-68(+) myeloid cells but not cancer cells in HNSCC patient tissues. (a) Cancer cells with high expression of *ATG7* did not show high expression of PD-L1 in HNSCC patient tissues. (b) In CD68(+) myeloid cells, *ATG7* was coexpressed with PD-L1. Representative IHC images from 3 HNSCC tumor tissues.

We have already revealed that *ATG7* was correlated with impaired immune function in HNSCC and mainly functioned in noncancer cells. We then investigated the cluster of noncancer cells influenced by ATG7. We mined 4 databases (TIMER, MCPcounter, EPIC, and CIBERSORT), and high *ATG7* expression was correlated with macrophages in 3 databases, dendritic cells in 2 databases, and CD8+T cells in 3 databases based on TCGA analysis (Figures 6(a)–6(d)). Furthermore, the expression of *ATG7* was mainly enriched in myeloid cells in GSE103322 (Figure 6(e)). The myeloid cells were reported to have adverse outcomes in cancer patients according to cancer types. Therefore, we further investigated the influence of myeloid cell proportion on overall survival in HNSCC through Cox regression models. A high proportion of myeloid cells was an adverse prognostic factor in the CIBERSORT (HR = 5.170*P* < 0.0001) and TIMER (HR = 27.2*P* < 0.0001) databases (Figure 6(f)). We next confirmed these correlations in HNSCC patients. The multiple immunofluorescence results showed that cancer cells with a high expression of *ATG7* did not have elevated expression of PD-L1, while CD68(+) myeloid cells had strong coexpression of *ATG7* and PD-L (Figures 7(a)–7(b)). Together, these data highlight a potential role for *ATG7* in myeloid cells, which may contribute to the immune suppressive microenvironment in HNSCC.

## 4. Discussion

The role of autophagy in cancer remains controversial. It is possible that both autophagy and autophagy-related genes play multifaceted roles depending on the tumor type and stage of the tumor. In HNSCC, Liu et al. revealed higher levels of cytoplasmic p62, suggesting impaired autophagy and its correlation with reduced overall and disease-specific survival [10]. However, elevated endogenous LC3-II expression has been reported in 90 oral cavity tumors, revealing the association of “high” levels of LC3-II with reduced overall survival. This supports the theory of autophagy reactivation during disease progression [22]. This uncertainty makes targeting autophagy for cancer therapy unpredictable. To overcome this challenge, it is important to determine the specific roles of different autophagy-related genes in both autophagy-dependent and -independent pathways. This understanding may contribute to precision autophagy-targeted therapies. In this research, we mined the TCGA database and found that *ATG9B* and *ATG7* had noncanonical autophagy-independent functions in HNSCC.

We found that ATG9B was a strong protective factor in HNSCC patients. ATG9B downregulation is significantly correlated with high EMT in HNSCC. By mining the HNSCC single-cell database, we further proved that *ATG9B* mainly functions in cancer cells through enhancing the epithelial phenotype of cancer cells. The correlation between autophagy and EMT is elusive [23]. On the one hand, cells are dependent on autophagy activation to survive during EMT. On the other hand, autophagy functions as a tumor-suppressive signal, which hinders the early phases of metastasis and activation of EMT [24]. It should be emphasized that in our verification in the single-cell database, *ATG9B* was not correlated with EMT markers (SNAIL, TWIST, and ZEB1), but did have a strong correlation with KRT family members. These results show that instead of influencing canonical EMT pathways like the WNT signaling pathway, *ATG9B* plays a more direct role in maintaining the epithelial phenotype of cancer cells. To our knowledge, there are no reports on *ATG9B* and cancer EMT, and further studies should focus on how *ATG9B* is involved in maintaining the epithelial phenotype of cancer cells instead of participating in EMT signaling pathways.

Another intriguing finding of our research was that *ATG7* had a strong correlation with the high expression of PD-L1 in TIME. Interestingly, *ATG7* seemed to function in myeloid cells instead of cancer cells in TIME. It is widely acknowledged that the upregulation of PD-L1 in cancer cells or upregulation of PD-1 or CTLA4 results in the immunosuppressive environment in TIME. However, it is easy to overlook that the increased expression of PD-L1 in immune cells can have the same effects. Fleming et al. found that mouse melanoma cells upregulated the expression of PD-L1 on mouse immature myeloid cells, leading to suppression of T-cell activation [25]. Zhang et al. specifically targeted PD-L1 in tumor-associated myeloid cells and showed a large synergistic curative effect with radiation therapy [26]. Increasing numbers of studies are focusing on the contribution of myeloid cell-derived PD-L1 instead of cancer-derived PD-L1 to the immunosuppressive environment in TIME [27, 28]. To our knowledge, there is only one study showing that *ATG7* was correlated with PD-L1 expression in bladder cancer cells [29]. Our findings preliminarily confirm that higher expression of *ATG7* was closely correlated with high PD-L1 expression in myeloid cells in HNSCC. The molecular mechanisms need to be further elucidated. Combined therapy targeting *ATG7* and PD-L1 in HNSCC may be a potential treatment.

There are also some limitations of the present study. Of particular, the correlation between the *ATG7* and PD-L1 expression in myeloid cells should be further verified using biocellular and biomolecular assays. Moreover, the therapeutical efficacy of the combination of ATG7-targeted agents and immune checkpoint blockade therapies can be further explored on multiple preclinical models.

Collectively, we preliminarily verified two noncanonical autophagy-independent functions of *ATG9B* and *ATG7* in HNSCC through the TCGA and single-cell databases. These findings increase understanding of the role of autophagy and autophagy genes in HNSCC and may contribute to precision autophagy-targeted therapies in HNSCC.

## Data Availability

The open-access datasets are available through the following URL: GSE103322 (https://www.ncbi.nlm.nih.gov/geo/query/acc.cgi?acc=GSE103322/) and the Cancer Genome Atlas (TCGA) HNSC project (http://xena.ucsc.edu/). All data generated or analyzed during this study are available from the corresponding author on reasonable request.

## Supplementary Materials

Supplementary Materials Figure S1: Expression of KRT genes in tumor and normal tissues in the TCGA-HNSCC cohort. Figure S2: ATG9B protein levels in human HNSCC tissues and paired adjacent normal tissues.
