# Supplementary material for: Unveiling the Noncanonical Autophagy-Independent Role of ATG7 and ATG9B in Head and Neck Squamous Cell Carcinoma (HNSCC)
Source: J Oncol. 2022 Oct 10;2022:9253938. doi: 10.1155/2022/9253938 (PMC9576406; doi:10.1155/2022/9253938)
Supplement: Supplementary Materials — Figure S1: Expression of KRT genes in tumor and normal tissues in the TCGA-HNSCC cohort. Figure S2: ATG9B protein levels in human HNSCC tissues and paired adjacent normal tissues. [file 9253938.f1.pdf]

Supplementary Figure 1

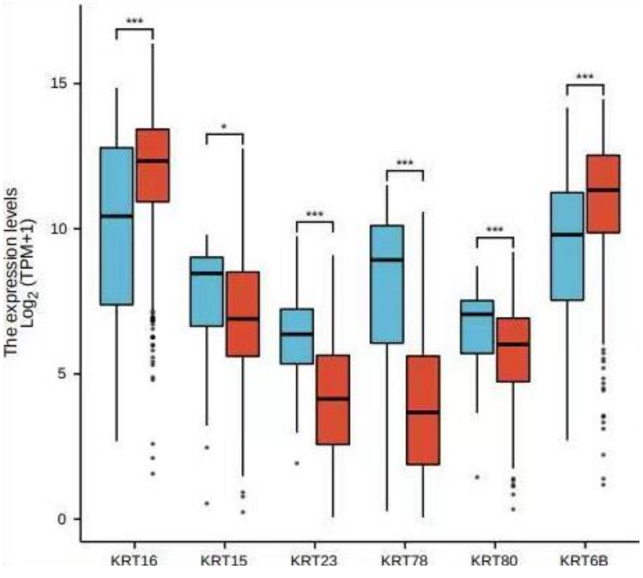

Supplementary Figure 1. Expression of KRT genes in tumor and normal tissues in HNSCC in TCGA.

Supplementary Figure 2

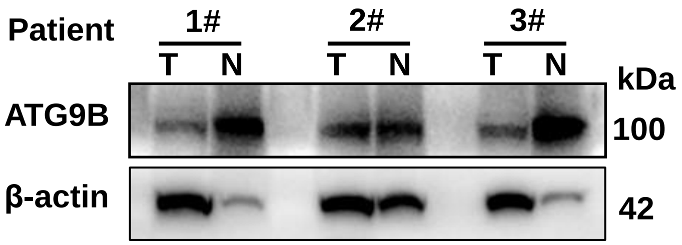

Supplementary Figure 2. ATG9B protein levels in human HNSCC tissues and paired adjacent normal tissues. “T” for tumor tissues and “N” for adjacent normal tissues.
